# An Updated Algorithm for the Generation of Neutral Landscapes by Spectral Synthesis

**DOI:** 10.1371/journal.pone.0017040

**Published:** 2011-02-15

**Authors:** Joseph D. Chipperfield, Calvin Dytham, Thomas Hovestadt

**Affiliations:** 1 Field Station Fabrikschleichach, Universität Würzburg, Rauhenebrach, Germany; 2 Department of Biology, University of York, Heslington, York, United Kingdom; 3 Muséum National d'Histoire Naturelle, Brunoy, France; University of South Florida College of Medicine, United States of America

## Abstract

**Background:**

Patterns that arise from an ecological process can be driven as much from the landscape over which the process is run as it is by some intrinsic properties of the process itself. The disentanglement of these effects is aided if it possible to run models of the process over artificial landscapes with controllable spatial properties. A number of different methods for the generation of so-called ‘neutral landscapes’ have been developed to provide just such a tool. Of these methods, a particular class that simulate fractional Brownian motion have shown particular promise. The existing methods of simulating fractional Brownian motion suffer from a number of problems however: they are often not easily generalisable to an arbitrary number of dimensions and produce outputs that can exhibit some undesirable artefacts.

**Methodology:**

We describe here an updated algorithm for the generation of neutral landscapes by fractional Brownian motion that do not display such undesirable properties. Using Monte Carlo simulation we assess the anisotropic properties of landscapes generated using the new algorithm described in this paper and compare it against a popular benchmark algorithm.

**Conclusion/Significance:**

The results show that the existing algorithm creates landscapes with values strongly correlated in the diagonal direction and that the new algorithm presented here corrects this artefact. A number of extensions of the algorithm described here are also highlighted: we describe how the algorithm can be employed to generate landscapes that display different properties in different dimensions and how they can be combined with an environmental gradient to produce landscapes that combine environmental variation at the local and macro scales.

## Introduction

Landscape ecology researchers assert that the spatial structure of the environment can play as much importance on key ecological outcomes, such as the survival and coexistence of species, than the biological characteristics of the species in question. It is not only the individuals, populations, or communities that have key ecological parameters of interest but also the landscape in which these entities operate [1]. Indeed the very idea of landscape ‘connectivity’, as described in [2] and [3], incorporates the effect of unequal distances between patches in the habitat matrix on metapopulation persistence (see also [4]).

Whilst the life history characteristics of a species, such as reproductive potential, mortality rate, and dispersal tendencies are undoubtedly linked to the habitats in which they reside, there has been some considerable effort amongst landscape ecologists to isolate the effects of the spatial and temporal configuration of these habitats on the long-term prospectus of phenomena of interest such as species ranges and patch-occupancy dynamics [5]–[7]. This desire has resulted in the development of so-called neutral landscape models (see [8]–[11]), which provides a mechanism for the generation of random landscapes with controllable spatial properties. Neutral landscapes can be used in models of ecological processes to allow for the assessment of the contribution of habitat matrix structure on the outcome of the ecological process being modelled. Such applications have borne critical results in the theory of species conservation and management: for example, [12] uses neutral landscapes models to identify a possible unimodal, but heavily skewed, relationship between species richness and the spatial heterogeneity of habitats. [13]–[16] all use neutral landscape models to help elucidate the effects of landscape structure on the evolution of dispersal and [17] uses a variation of the same neutral landscape model to produce ‘disturbance landscapes’ to assess the response of population dynamics to the autocorrelation of disturbance events.

Neutral landscape models were initially conceived to provide simple binary grids of suitable and unsuitable habitat types. In these models, a habitat abundance parameter, 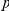, determines the proportion of the landscape that is suitable. A simple algorithm to simulate such landscapes would involve starting with an entirely unsuitable landscape and iteratively selecting a random cell from the pool of unsuitable habitat types and classifying it as suitable until the correct proportion of suitable cells is achieved. These ‘percolation models’ have been employed successfully in a number of studies ([8], [18], [19] for example) but are limited in that it is not possible for the users of these models to control explicitly the spatial autocorrelation of the outputs. [20] and [21] develop these models further, using fractal curdling to allow for a spatial hierarchy in the assignment of suitable patches. Here, occupancy is assigned at successively fine-scale spatial resolutions with each hierarchical division. At the 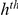 hierarchy, a 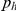 proportion of cells are assigned as habitable from each of the cells assigned as habitable from the previous hierarchical allocation. The total proportion of habitable cells at the end of the allocation is therefore the product of the allocation probabilities at each spatial resolution such that 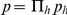.

**Figure 1:**
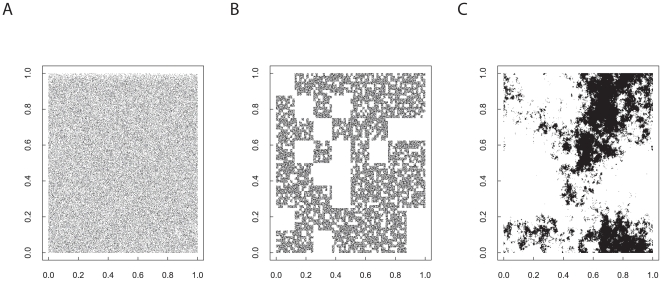
Realisation of three cellular lattice landscapes (512 × 512) with the same proportion of suitable habitat (73728 cells ca 28%) but generated using different neutral models (after [33]). Black cells denote suitable habitat. Figure A depicts a landscape generated by selecting the relevant proportion of cells at random and assigning suitability to these cells. Figure B is a landscape generated by hierarchical curdling using three stages. In the first stage, the landscape is divided into 64
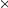
64 size tiles and then 75% of these tiles are selected as containing suitable habitat. The second stage of the process involves dividing the selected tiles from the first stage and dividing them into 8
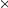
8 tiles. 75% of the 8
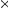
8 tiles are selected in each of the coarser resolution tiles selected in the previous stage. The final stage selects 50% of the cells contained in the selected tiles of the previous stage and assigns them as suitable habitat. Figure C shows a landscape generated by fractional Brownian motion with a Hurst exponent of 0.2 using the algorithm outlined in this manuscript. The continuous output from this process is divided into suitable and non-suitable habitat by ranking the cell values and selecting the top 73728 as suitable.

**Figure 2:**
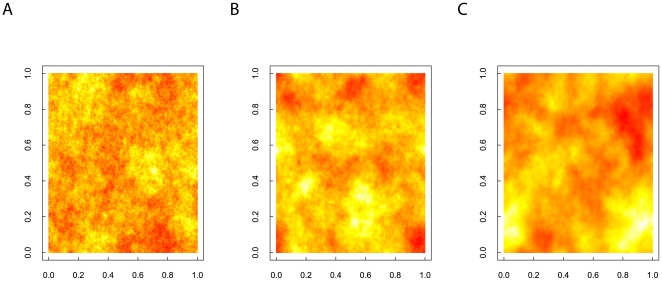
Three realisations of landscapes (256 × 256 cells) created by the spectral synthesis algorithm for fractional Brownian motion. Figure A is generated using a Hurst exponent of 0.1, figure B uses a Hurst exponent of 0.5, and finally, 0.9 is used as the Hurst exponent in the generating mechanism for figure C.

It is not always adequate to represent landscapes as a simple surface of binary habitat values however. Instead it may be preferable in some situations to allow the species being modelled to respond to a continuum of habitat values. Realisations of this, more general, representation of habitat structure can be achieved by using one of a number of algorithms that simulate fractional Brownian motion (*sensu* [22]). These methods have the advantage that they produce autocorrelated landscapes controllable by a single parameter, the Hurst exponent, 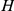 (see [23]). Landscapes created using this method can exhibit quite different spatial characteristics than those generated using random or hierarchical random methods, providing landscapes with a more natural-looking aggregation of suitable habitat types (see figure 1). As 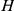 tends towards zero, the landscapes become more heterogeneous, whilst values close to one become more self-similar. Figure 2 shows a series of landscapes generated using different Hurst exponents.

The two most commonly employed algorithms used in the simulation of fractional Brownian motion are midpoint displacement methods [23] and spectral synthesis methods [23], [24]. Midpoint displacement algorithms are easily implemented and, as such, have seen wide popularity in the application of computer graphics. They do suffer from a number of drawbacks however: firstly, midpoint displacement algorithms do not produce realisations of true fractional Brownian motion when the Hurst exponent is not set equal to 0.5 [25]. For many applications it is desirable that the fractal surface exhibits no gradients in habitat values, either because the study requires that the effects of spatial autocorrelation on ecological phenomena be tested in isolation from the effects of a gradient, or because a gradient with attributes under control of the investigator are to be added to the random fractal surface. From [23] it is shown that the midpoint displacement algorithm, as commonly implemented, produces non-stationary landscapes. Moreover, whilst [23] describe a one and two dimensional implementation of midpoint displacement, the algorithm becomes unwieldy when extended to higher dimensions.

In contrast, spectral synthesis algorithms produce stationary landscapes that better approximate true fractional Brownian motion. One and two dimensional versions of the spectral synthesis algorithm appears in [23] whilst [24] provides a more general algorithm for an arbitrary number of dimensions. However, the algorithm as proposed in the appendix of [24] can produce landscapes that have values that are correlated with the distance from the origin of the dimensional coordinates of the cell. This clearly undesirable artefact of the algorithm produces a distinct visual ‘smearing’ on two dimensional landscapes and a tendency for similar values to occur in diagonal neighbourhoods. Here we outline an updated version of the algorithm described by [24], allowing the spectral synthesis methods to be employed in the generation of landscapes of an arbitrary number of dimensions but with the artifacts that have appeared in previous incarnations removed. We show how this extension of the algorithm can be useful in generating dynamic landscapes in spatio-temporal studies, allowing not only investigator control of spatial autocorrelation but also the degree of habitat ephemerality: the autocorrelation of habitat in time.

## Methods

### Spectral Synthesis Algorithms

Spectral synthesis methods involve the generation of random Fourier coefficients with the restriction that the spectral density (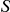) of the vector of frequencies in each dimension, 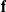, must satisfy the following condition:
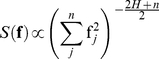
(1)where 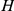 is the Hurst exponent and 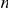 is the number of dimensions. Once the Fourier coefficients are generated, it is a simple case to perform an inverse Fourier transform to convert the spectral densities into landscape values with the appropriate spatial properties. There are a number of algorithms that can perform an inverse multidimensional Fourier transform such as the fft routine implemented in the R statistical package, the fourn algorithm of [26], or the FFTW numerical library of [27]. Where the different spectral synthesis methods differ is in how they generate these Fourier coefficients. The appendix of [24] describes one such algorithm for the creation of these Fourier coefficients. Here we include the multidimensional case in full to help illustrate the difference between the algorithm of [24] and our own.

*Algorithm 1*

1. Initialise an 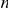-dimensional array, 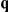, with 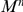 complex elements where 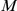 is the length of the landscape to be generated in each dimension. 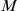 must be a power of two.
2. For each 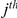 element of array 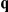:
  - Calculate the vector of dimensional coordinates, 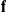, of cell 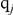.
  - Generate an amplitude, 
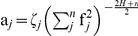
, where 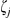 is a number drawn from a standard normal distribution.
  - Generate a random phase, 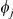, by drawing a value from a uniform distribution defined between 0 and 2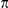.
  - Set the element 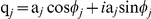.
3. Take the multidimensional inverse Fourier transform of the array 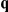.
4. Use the real valued output from the Fourier transform as values for the landscape output.

The output we desire is real valued. To generate real-valued output from the inverse Fourier transform we must ensure that the complex coefficients adhere to a conjugate symmetry condition (see page 109 of [23]) whereby each element of the coefficients vector at frequency coordinates 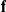, 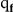, is the complex conjugate at vector element with symmetric frequency coordinates 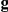, namely
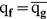
(2)where each element of vector 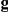 is defined by
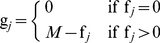
(3)


The algorithm of [24] does not account for this conjugate symmetry requirement and, as a result, the dropping of the complex component in the last step creates landscapes with cell values correlated with their dimensional coordinates. We suggest here employing a multidimensional extension of the SpectralSynthesisFM2D algorithm described in [23]. This extension provides an algorithm that satisfies the conjugate symmetry requirement of [23] whilst also maintaining generality for the creation of multidimensional landscapes:

*Algorithm 2*

1. Initialise a multidimensional array, 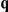, with 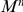 complex elements where 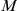 is the length of the landscape to be generated in each dimension. 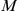 must be power of two.
2. For each 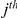 element of array 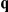:
  - Calculate the vector of dimensional coordinates, 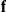, of cell 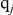.
  - Calculate the vector of symmetric dimensional coordinates, 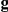, using equation 3.
  - Generate an amplitude, 
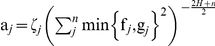
, where 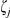 is a number drawn from a standard normal distribution.
  - Generate a random phase, 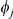, by drawing a value from a uniform distribution defined between 0 and 2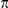.
  - Set the element 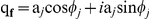.
  - Set the element 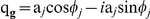.
  - If all coordinates of the vector, 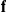, are equal to either zero or 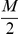, then set the imaginary component of 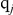 to zero.
  - If all coordinates of the vector, 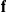, are equal to zero, then set the real component of 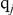 to zero.
3. Take the multidimensional inverse Fourier transform of the array 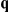.
4. Use the real valued output from the Fourier transform as values for the landscape output.

The extra calculation taken at step 2 of the above algorithm ensures that the conjugate symmetry condition is met and that the artifacts of the [24] algorithm are avoided. As a result the imaginary component of the output vector after the Fourier transform in algorithm 2 will be zero (or a very close value to it owing to the approximate floating number arithmetic) and, unlike the first algorithm, the dropping of the imaginary part will therefore not represent a loss of information.

The inverse Fourier transform process scales the spectral coefficients according to the length of the landscape in each dimension. Unequal length in each dimension can therefore result in differing levels of autocorrelation in each direction and, as a result, we restrict the description of the algorithm to the case where these lengths are equal. Lengths restricted to a power of two also allow an efficient Fourier transform and so we have also placed this extra restriction on the value of 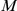. However, landscapes of differing sizes can be easily created by generating a landscapes with the smallest value of 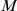 that is equal to or larger than the largest dimensional extent of the desired landscape and then using the first set of elements in each dimension. This may appear inefficient at first but, in most cases, it is quicker to do this rather than to only generate the minimal number of spectral coefficients required in the spectral synthesis phase of the algorithm and employ a less efficient Fourier transform algorithm to convert these coefficients to the real-valued output.

An implementation of this algorithm for the statistical computing platform R, and instructions to use the supplied functions, can be found in the ecomodtools package hosted on the R-Forge repository. To install the package from the R console simply type the following command whilst connected to the internet: install.packages(“ecomodtools”, repos = “ http://R-Forge.R-project.org ”). This package is open source and released under the General Public License (version 2 or later).

### Monte Carlo Testing

The spatial artefacts induced by the algorithm of [24] can be described in terms of the anisotropic patterns in the surfaces that they produce. In this paper, we have employed Monte Carlo simulation to test the isotropic properties of 300 landscapes generated using the algorithm of [24] (algorithm 1) and 300 landscapes created using the updated algorithm of [23] described in the methods section of this paper (algorithm 2). For each of the 300 landscapes generated using each synthesis algorithm, one of three Hurst exponents was used to control the spatial self-similarity of the landscape: 0.1, 0.5, or 0.9. This resulted in a total of 600 landscapes generated with 100 landscapes once divided between each synthesis algorithm and Hurst exponent combination. Each landscape is generated with a total of 1024 cells occupying a 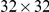 grid.

**Figure 3:**
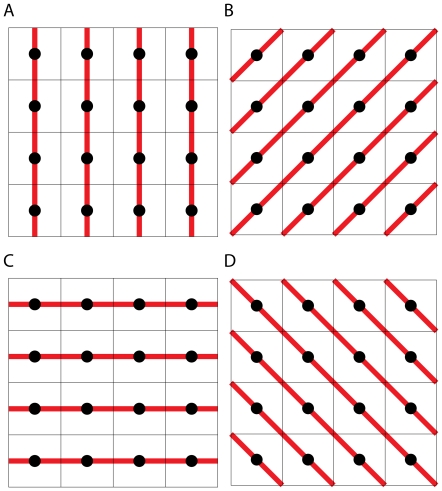
Illustration of neighbourhood definitions used in construction of the spatial autocorrelograms used in this study. Figures A–D display networks of cells connected with links running at angles of 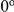, 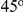, 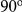, and 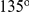 clockwise from the 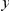-axis respectively. The boundaries of the network in each spatial dimension are wrapped around to form a torus. Red lines denote the network links between the cell centres.

**Figure 4:**
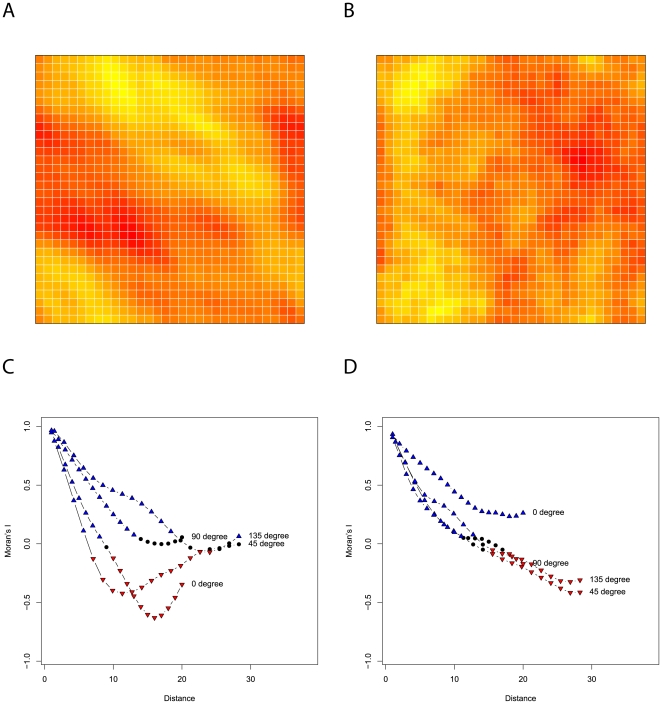
One realisation of landscapes generated using each of the two algorithms described in this paper with accompanying directional autocorrelograms. Figures A and B are realisations of a 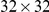 landscape generated by employing algorithms 1 [24] and 2 (adapted from [23]) respectively. Both landscapes have been generated using a Hurst exponent of 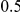. Figures C and D are the accompanying directional autocorrelograms to figures A and B respectively with Moran's 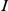 calculated over each of the four networks shown in figure 3. Blue triangles represent values of Moran's 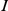 that are significantly larger (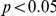) than what would be expected in a random landscape Red inverted triangles denote values of Moran's 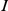 significantly smaller (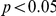) than what would be expected in a random landscape.

One method through which the anisotropy of a landscape can be assessed is by the comparison of directional spatial autocorrelograms [28]. Here, we define a neighbourhood of connectivity restricted to groups of cells with centres that fall directly on the line at an angle, 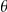, measured clockwise from the 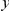-axis. We use four values for 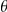: 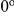, 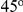, 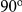, and 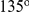. This creates four networks with cells connected only on the vertical, bottom-left to upper-right diagonal, horizontal, and bottom-right to upper-left diagonal adjacent cells respectively (see figure 3). Spectral synthesis algorithms produce landscapes that are periodic, and we incorporate this feature into the analysis by wrapping the links at the boundaries of the connectivity network so that the network is defined on a torus. Autocorrelation for each distance 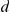 is assessed by calculating Moran's 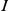 [29] over all pairs of cells with centres exactly 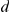 units apart with distance measured along the links of the network. For any distance class, Moran's 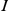 takes values greater than zero (usually less than one) when the data exhibit positive autocorrelation and values less than zero (usually greater than negative one) when the data exhibit negative autocorrelation. If we define 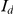 as the value of Moran's 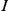 for distance class 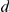 then
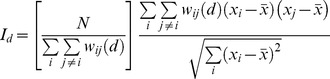
(4)where 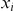 is the 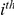 value of 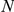 data values. 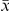 is the mean data value and 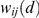 is a weighting function taking the distance between the cell centroids as input. Here we use a simple identity function for 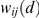 which equals one when the centroids of the 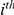 and 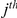 cells are exactly a distance, 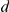, apart. 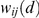 is equal to zero at all other times. Figure 4 shows one realisation from each of the algorithms described in this paper with an accompanying autocorrelogram.

To aggregate the results of each of the 100 landscapes generated in each factor combination, we calculate two difference statistics, 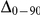 and 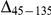 of Moran's 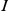, which describe the difference between the Moran's 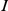 statistic calculated on the 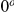 and 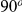 neighbourhoods and the difference between the Moran's 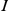 statistic calculated on the 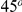 and 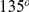 neighbourhoods respectively. These statistics describe the difference in autocorrelation of the landscapes measured in perpendicular directions, which for 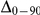 Moran's 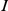, runs parallel to the cardinal axes, whilst for 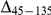 Moran's 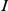, runs parallel to the intercardinal axes. Positive values for 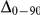 Moran's 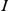 occur in landscapes with higher autocorrelation in the north-south direction than in the east-west direction whilst negative values for 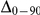 Moran's 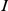 denotes landscapes with lower autocorrelation in a north-south direction then in an east-west direction. Similarly, positive values for 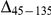 Moran's 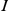 denote landscapes with higher autocorrelation measured along the direction running from the south-west to the north-east than in the perpendicular direction running from the south-east to the north-west. The reverse is also true for negative values of 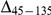 Moran's 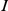. Values close to zero for both statistics are representative of landscapes that exhibit isotropic behaviour in the directions tested whilst values of large magnitude for either statistic denote landscapes with very different levels of autocorrelation when measured parallel to the relevant perpendicular axes.

## Results and Discussion

### Anisotropy Analysis

**Figure 5:**
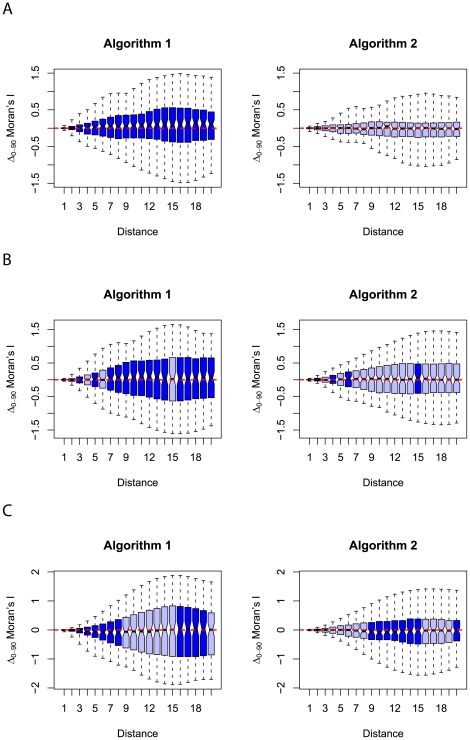
Series of box plots showing the difference in autocorrelation measured using Moran's 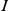 along lines parallel to the two cardinal axes. Figure A displays the results for landscapes generated by employing both synthesis algorithms with a Hurst exponent of 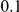 (high heterogeneity). Figure B displays similar results for landscapes generated with a Hurst exponent of 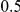 (intermediate heterogeneity) whilst figure C shows results for landscapes generated using a Hurst exponent of 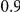 (low heterogeneity). The dashed red line shows the location of no difference between the autocorrelation measured in each axis direction (where 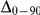 Moran's 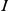 is zero) and represents the expected median for a series of isotropic landscapes. Lighter coloured boxes show the inter-quartile range of the results from a synthesis algorithm with a median of 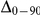 Moran's 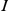 closer to zero at the respective distance class than the alternative algorithm. Conversely, darker coloured boxes indicate that the magnitude of the median value of 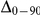 for a given synthesis algorithm exceeds that exhibited by landscapes generated using the alternative. The notches of the box plots extend from the median to 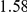 multiplied by the inter-quartile range divided by the square root of the sample size (in this case 100) representing a rough 95% confidence interval for the median based on asymptotic normality [35]. The box whiskers extend to the full range of data values.

**Figure 6:**
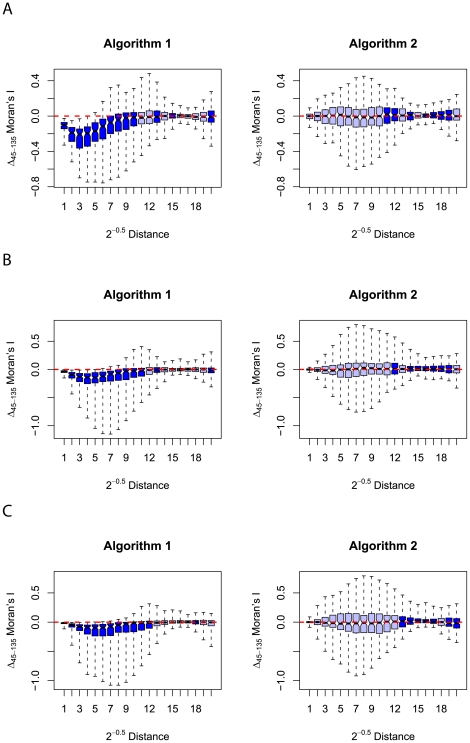
Series of box plots showing the difference in autocorrelation measured using Moran's 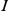 along lines parallel to the two intercardinal axes. Figure A displays the results for landscapes generated by employing both synthesis algorithms with a Hurst exponent of 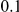 (high heterogeneity). Figure B displays similar results for landscapes generated with a Hurst exponent of 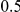 (intermediate heterogeneity) whilst figure C shows results for landscapes generated using a Hurst exponent of 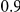 (low heterogeneity). The dashed red line shows the location of no difference between the autocorrelation measured in each axis direction (where 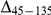 Moran's 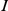 is zero) and represents the expected median for a series of isotropic landscapes. Lighter coloured boxes show the inter-quartile range of the results from a synthesis algorithm with a median of 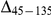 Moran's 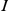 closer to zero at the respective distance class than the alternative algorithm. Conversely, darker coloured boxes indicate that the magnitude of the median value of 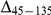 for a given synthesis algorithm exceeds that exhibited by landscapes generated using the alternative. The notches of the box plots extend from the median to 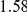 multiplied by the inter-quartile range divided by the square root of the sample size (in this case 100) representing a rough 95% confidence interval for the median based on asymptotic normality [35]. The box whiskers extend to the full range of data values.

Figure 5 shows a series of box plots for the 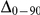 Moran's 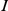 statistic calculated for each set of landscapes generated using the different Hurst exponents and synthesis algorithms. Figure 6 shows similar plots for autocorrelation measured parallel to the intercardinal axes (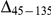 Moran's 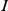).

From figures 5 and 6 we can see that there is a marked tendency for smaller magnitudes in the medians of 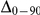 and 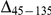 of Moran's 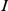 from the set of landscapes generated using the updated algorithm of [23] described in this paper. This effect is present in both the cardinal and intercardinal comparisons and is most prevalent in landscapes generated using a lower Hurst exponent. This suggests that, for mid-to-low range values of the Hurst exponent, then, on average, algorithm 2 produces landscapes that exhibit fewer anisotropic characteristics than those generated using algorithm 1. For landscapes generated using the highest Hurst exponent tested in this analysis (0.9) the relative performance of the algorithms is less clear and no one algorithm produces a consistently lower magnitude of directional autocorrelation disparity across all distance classes.

For comparisons of autocorrelation in directions parallel to the cardinal axes (figure 5), we see that both synthesis algorithms produce a series of landscapes with values of 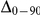 Moran's 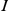 centred around zero but the range of values taken from landscapes generated using the first synthesis algorithm is much larger. These results reveal no general differences between the autocorrelative properties measured in either cardinal direction for either algorithm but that individual landscapes generated using the first algorithm can potentially exhibit a greater range of anisotropic properties than those generated using the second algorithm.

The variance in the results that we have seen here, where values for 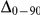 Moran's 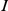 lie around zero but with a fair degree of spread in either direction, can arise from an algorithm that simulates an isotropic process but for which the variance represents deviations from isotropy for each individual realisation. Alternatively, the algorithm may simulate a truly anisotropic process but with the anisotropy exhibited in a non-cardinal direction. In this instance, each realisation would produce an anisotropic landscape with the direction of maximum autocorrelation being a random realisation of a direction set around the mean anisotropy direction of the process being simulated. These deviations, when large, may also be exhibited in the directional autocorrelograms defined on networks with neighbourhoods that do not run parallel to the direction being tested. If the directionality of the process being simulated by the algorithm falls directly between the cardinal axes, then the variance of realisations of this process can produce values of 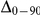 Moran's 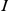 of large magnitude and therefore increase the variance of the autocorrelograms measured parallel to the cardinal axes. This latter, anisotropic, explanation is supported as the generating mechanism for the variance of 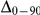 Moran's 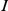 in the landscapes of synthesis algorithm 1 is supported by the visual ‘smearing’ present on these landscapes (see figure 4) and by the indubitable non-zero central tendency of 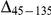 Moran's 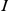 exhibited in the proximal distance classes.

In contrast to the anisotropic properties of algorithm 1, the distributions of both 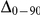 and 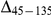 Moran's 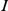 of landscapes generated using algorithm 2 are centred around zero. These results, in addition to the relatively artefact free appearance of the landscapes, such as the landscape realisation of 4, suggests that algorithm 2 accurately simulates a process with isotropic properties. Whilst it is feasible that algorithm 2 simulates an anisotropic process with direction of maximum autocorrelation that does not follow any of the directions tested here, it is unlikely that such directionality would not be evident in the autocorrelogram difference statistics in either the cardinal or ordinal directions. Therefore, a reasonable interpretation of the results presented in figures 5 and 6 is that the updated algorithm of [23] described in this paper successfully corrects the artefact of intercardinal anisotropy present in the algorithm of [24].

### Further Extensions

Landscapes generated by spectral synthesis have the property that they are periodic. For some applications this is not necessarily an undesirable property. Many theoretical simulation studies, such as that of [30] and [13], use periodic boundary conditions to avoid some of the artifacts that other boundaries conditions can induce. If the landscape over which the ecological simulation is also periodic then it avoids individuals experiencing sharp discontinuities as they cross the boundaries of the simulation arena. If periodicity is not desired then this property is easily assuaged by constructing landscapes larger than required and then using a fraction of the total surface area. To avoid periodic effects, [24] suggests generating surfaces two to four times larger than the landscape to be employed and selecting only the first set of cells in each dimension that comprises the desired volume.

**Figure 7:**
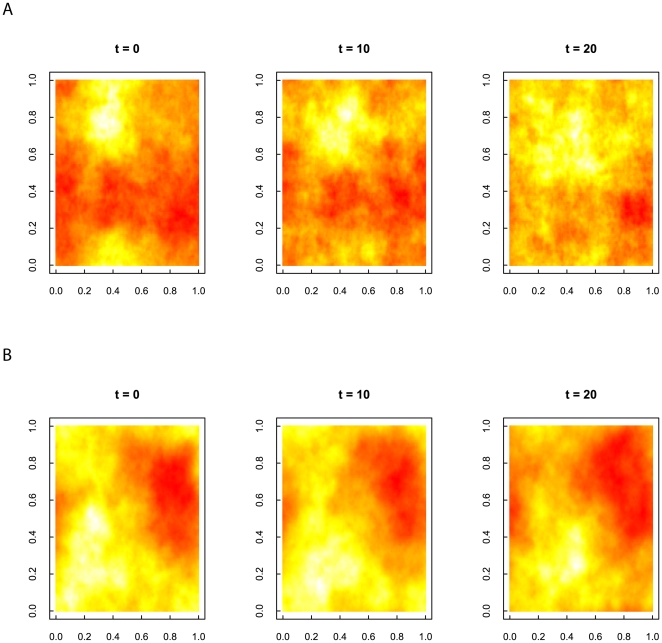
Three samples, taken at times 0, 10, and 20, from two time series of landscapes (128 ×128 ×128) generated using the spectral synthesis algorithm described in this paper. Both landscapes are generated using Hurst exponents of 0.9 in the spatial dimension but figure A uses a temporal Hurst of 0.1 and figure B uses a temporal Hurst of 0.9.

Whilst we have viewed the anisotropy present in algorithm 1 as an unwanted artefact, there are times when it is usual to specify different levels of autocorrelation in each dimension. [24] describes an extension of the standard spectral synthesis algorithm to do just this. This is particularly useful in the generation of dynamic landscapes in spatio-temporal studies as it allows separate ascription of spatial and temporal autocorrelation. Figure 7 shows two examples of landscapes generated with direction-dependent scaling. This extension requires very little change to the algorithm described above; the overall Hurst exponent, 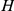, becomes a function of the set of dimension-specific Hurst exponents
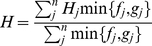
(5)where 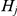 is the Hurst exponent of the 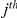 dimension. Effectively the Hurst exponent used in the two algorithms is replaced by a ‘composite Hurst exponent’, equivalent to an average of the dimension-specific Hurst exponents weighted according to the frequency coordinates in each dimension. This composite Hurst exponent must be recalculated for every spectral coefficient generated. It is important to make clear that whilst this composite Hurst exponent changes value for each element, the dimension-specific Hurst exponents do not and, as such, the underlying autocorrelative properties of the generated landscape do not change with the dimensional coordinates.

**Figure 8:**
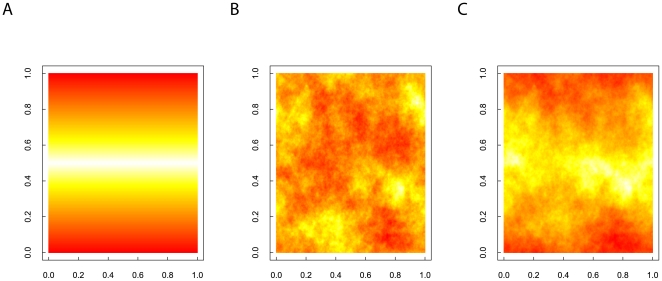
Combining an environmental gradient surface with local environmental heterogeneity to provide a composite landscape (see [31]). Figure A illustrates a simple gradient landscape (256
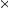
256) with each cell value set to one minus 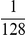 multiplied by the absolute distance (in number of cells) of the y-coordinate of the cell centre from the middle of the landscape. This provides a piecewise linear gradient with values bounded between zero and one symmetrically decreasing from the centre of the landscape. Figure B illustrates a fractal landscape (256
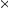
256), generated using the spectral synthesis algorithm described in this paper with a Hurst exponent of 0.5, and normalised so that all values fall between the range of zero and one. Figure C shows a simple combination of these landscapes, 0.5 multiplied by the values of gradient landscape plus 0.5 multiplied by the values of the fractal landscape, to produce another landscape with values still bounded between zero and one.

Specifying direction-specific Hurst exponents to produce anisotropic landscapes may usefully characterise some environments but there are times when it is also necessary to model non-stationary landscapes. The documentation and testing of the effect of environmental gradients on various ecological phenomena is an area of active research. However, within any environmental gradient there is likely to be many local sources of spatially correlated environmental variability. To capture this process, [31] suggest the use of linear combinations of gradient surfaces with a neutral landscape model to jointly model the effect of gradients with local variability. Figure 8 shows an example of these composite landscapes.

### Neutral Landscapes and Conservation Ecology

The spatial configuration of habitats is an important factor in the survival and long-term viability of populations [1]. Given the importance of habitat configuration in conservation biology, it is imperative we continue the develop the theory that underlies metapopulaton dynamics. One method to develop and test theories of landscape ecology is to run field experiments. However, assessing the effects of habitat configuration with field experiments can be problematic. Robust statistical analysis of spatial structure requires replicates of landscapes with similar spatial characteristics and a wide variety of landscape configurations which can result in experiments that are costly to perform. Given the relative ease of performing simulation studies, we can supplement field studies with models of population dynamics run over landscapes generated using neutral landscape models [14]. This provides a much simpler and cost-effective way to develop theory in the field of landscape ecology.

Real landscapes are not ideal fractals [32], but they often exhibit fractal-like properties that can make modelling them as such sufficient for the purposes of theoretical investigation or hypothesis generation [33], [34]. Environmental gradients in nature are also never smooth and the hybrid approach described by [31] provides an elegant way to assess how local environmental variability can interact with a macro-scale gradient to produce patterns observed in nature. Given the ever-prevalent nature of gradients in ecological and evolutionary research, the existence of tools such as the one described in this paper can be critical in aiding the exploration of ideas and forwarding our understanding.

We have presented here a potentially valuable tool for use in simulation-based ecological investigations. By isolating the effects of landscape structure, we are given a method by which to assess how spatial and temporal autocorrelation affect ecological processes. We have shown how landscapes generated using the updated algorithm described in this paper do not exhibit many of the artefacts displayed in the products in earlier algorithm incarnations. This paper also describes a number of developments of the standard neutral landscape model which greatly expands the number of possible uses of these models.

## Supporting Information

Document S1 A translation of the article abstract into German.
(PDF)
