# Supplementary material for: An Updated Algorithm for the Generation of Neutral Landscapes by Spectral Synthesis
Source: PLoS One. 2011 Feb 15;6(2):e17040. doi: 10.1371/journal.pone.0017040 (PMC3039648; doi:10.1371/journal.pone.0017040)
Supplement: Document S1 — A translation of the article abstract into German. (PDF) [file pone.0017040.s001.pdf]

Ökologischen Muster im Raum und Zeit können sowohl durch inherente Eigenschaften ökologischen Prozesse, als auch durch die Landschaft, in der diese Prozesse stattfinden, verursacht werden. Eine Möglichkeit diese beiden Effekte zu trennen besteht darin Modelle ökologischer Prozesse in Landschaften mit kontrollierten räumlichen Eigenschaften ablaufen zu lassen. Eine Reihe von Verfahren wurden entwickelt um sogenannte 'neutral landscapes' zu erzeugen, darunter einige vielversprechende, die sich aus der Simulation von fraktionellen Brown'schen Molekularbewegungen ableiten. Entsprechende Verfahren weisen bisher jedoch einige Defizite auf: einerseits können sie nicht leicht auf mehrere Dimensionen erweitert werden, anderseits erzeugen sie unerwünschte Artefakte. Wir stellen hier einen verbesserten Algorithmus zur Erzeugung von neutral landscapes vor, der diese Probleme beseitigt. Wir untersuchen das Ausmaß von unerwünschter Anisotropie in Landschaften, die mit unserem verbesserten Algorithmus erzeugt wurden, und vergleichen diese mit entsprechenden Ergebnissen für Landschaften, die mit Hilfe eines häufig verwendeten Standardalgorithmus erzeugt wurden. Während letzterer deutliche Anisotropie verursacht (diagonale Streifen), tauch das Problem bei unserem neuen Algorithmus nicht auf. Darüber hinaus zeigen wir, wie mit Hilfe des Algorithmus auch Landschafen mit unterschiedlichen Eigenschaften in unterschiedlichen Dimensionen erzeugt werden können. Der Algorithmus erlaubt auch die Kombination von neutral landscapes mit Gradienten und damit die Erzeugung von Landschaften, die Umweltvarianz über verschiedene Skalen kombinieren.
